# A Sense of Coherence Approach to Improving Patient Experience Using Information Infrastructure Modeling: Design Science Research

**DOI:** 10.2196/35418

**Published:** 2022-04-12

**Authors:** Patricia A H Williams, Brendan Lovelock, Javier Antonio Cabarrus

**Affiliations:** 1 Flinders-Cisco Digital Health Design Lab, Flinders Digital Health Research Centre College of Science and Engineering Flinders University Adelaide Australia; 2 College of Business and Law RMIT University Melbourne Australia; 3 HammondCare Sydney Australia

**Keywords:** medical informatics, information infrastructure, digital hospitals, patient experience, implementation, eHealth

## Abstract

**Background:**

Health care provider organizations are complex and dynamic environments. Consequently, how the physical and social environment of such organizations interact with an individual is a primary driver of an individual’s experience. Increasingly, the capabilities required for them to successfully interact with those within their care are critically dependent on the information infrastructure they have in place, which enables people, both patients and staff, to work optimally together to deliver their clinical and operational objectives.

**Objective:**

This study aims to design a framework to address the challenge of how to assemble information systems in health care to support an improved sense of coherence for patients, as well as potentially innovate patients’ experiences, by connecting and orchestrating the synergy among people, processes, and systems.

**Methods:**

It is necessary to understand the needs of health care providers and patients to address this challenge at a level relevant to information process design and technology development. This paper describes the design science research method used to combine the sense of coherence, which is a core concept within the Antonosky salutogenic approach to health and well-being, with an established information infrastructure maturity framework, demonstrating the coalescence of 2 distinct conceptual perspectives on care delivery. This paper provides an approach to defining a positive and supportive health care experience and linking this to the capabilities of an information- and technology-enabled environment.

**Results:**

This research delivers a methodology for describing the patient experience in a form relevant to information infrastructure design, articulating a pathway from information infrastructure to patient experience. It proposes that patient experience can be viewed pragmatically in terms of the established sense of coherence concept, with its ability to identify and guide resources to modulate a patient’s environmental stressors. This research establishes a framework for determining and optimizing the capability of a facility’s information infrastructure to support the sense of coherence defined by the experiences of its patients.

**Conclusions:**

This groundbreaking research provides a framework for health care provider organizations to understand and assess the ability of their information infrastructure to support and improve the patient experience. The tool assists providers in defining their technology-dependent operational goals around patient experience and, consequently, in identifying the information capabilities needed to support these goals. The results demonstrate how a fundamental shift in thinking about the use of information infrastructure can transform the patient experience. This study details an approach to describing information infrastructure within an experience-oriented framework that enables the impact of technology on experience to be designed explicitly. The contribution to knowledge is a new perspective on modeling how information infrastructure can contribute to supportive health-promoting environments. Furthermore, it may significantly affect the design and deployment of future digital infrastructures in health care.

## Introduction

### Background

Information technology (IT) is increasingly playing a pivotal role in forming an individual’s experience within a health system. The way processes are initiated and delivered and how we communicate our choices and needs, even the light and climate in the room, are now mostly interfaced and controlled through IT. Although there has been extensive work on defining an organization’s ability to deliver such technologies [1], there is a scarcity of information on how technology influences the experiences of staff, patients, and families in the health systems.

The challenge is to construct health care environments that provide enhanced patient experience while enabling high-quality, accessible, and efficient care. To address this, it is necessary to understand more precisely how information and information systems affect patient and staff experiences both now and in the future.

Experience is a complex concept. Its realization is contextual to a person’s environment, current emotional state, past experiences, and future expectations. In health care, the question has always been how to define a good experience and identify the elements of that experience that the hospital and its services can contribute. Significant survey-based work has been conducted to isolate the factors that patients believe contribute to a positive experience. Indeed, most care delivery organizations conduct detailed surveys after an episode of care to understand how well patient needs and expectations are met [2].

However, to date, there has been little research on why patients identify these factors. Understanding the forces driving patients’ preferences would provide a clearer picture of how to change the hospital environment to improve the perceived experience. Consequently, we need improved models to shape experiences in health care. These models would include health care system–controlled factors that drive experience and describe how these factors come together.

This research aims to design a framework to address the challenge of how to assemble information systems in health care to support an improved sense of coherence (SOC) for patients, as well as potentially innovate patients’ experiences, by connecting and orchestrating the synergy among people, processes, and systems. The resulting framework provides a method for shaping the patient experience through the improved use of a hospital’s digital infrastructure and for the assessment of the maturity of this use based on an established digital infrastructure assessment methodology [3].

### Literature Review

To clarify the relationship between experience and technology in health care, it is important that our definition of a health care experience is appropriate and that a model for generating experience is established. Through this model, the relationship with technology can be detailed. Health care has adopted a specific definition for experience, in which the actions that generate interactions between a patient and their operational environment (people, place, or process) are defined as the experience, and an individual’s personal response to those actions is defined as satisfaction [4,5]. Both of these interrelated elements need to be accounted for when considering concepts such as the SOC to account for the generation of experience. In this review, we look at the definition of patient experience and patient satisfaction separately before bringing them together through the lens of SOC and relating them to information infrastructure through the infrastructure assessment methodology.

### Patient Experience

Patient experience has been used to identify the weaknesses and strengths of health care delivery, with a view to driving quality health care improvements and promoting patient choice [6]. Such measures can report on communication and, more importantly, the patient’s experience related to their involvement in their own care decisions [7]. Hence, it provides both a utilitarian feedback loop on health care delivery processes and the measurement of humanistic characteristics experienced by patients during their episodes of care.

Studies have found positive associations between patient experience and improved health outcomes, often through improved health care delivery processes [6,8-11]. Despite this, there is no consistent agreement about the quality outcomes, despite patient experience being considered a complementary measure for quality [9,12]. Indeed, several systematic reviews have examined patient experience and an elusive search for a specific definition [6,12-14].

### Patient Satisfaction

Patient satisfaction can measure three things [15]: sufficiency of treatment and care received to improve health outcomes; fulfillment of requests from patients and families for treatments and diagnostics that are not clinically needed and may be harmful; and person-centered well-being factors such as communication, dignity, and respect and the associated logistical factors such as ease of making appointments, accessible parking, hospital physical environment and location, and hospital gowns. Arguably, the latter aspects relate to well-being and are intrinsically linked to a personal sense of worth [16]. The high variability of patient satisfaction can be confounded by non–clinically related factors [9,17], over which the health care team has no control [18].

There are many dimensions to health care and, therefore, how a patient experiences an episode of care; consequently, how satisfied they are with the experience is one measure [19]. However, it is a measure that is variable for everyone and means something different for each person. Worryingly, research has shown that patient satisfaction scoring can have a negative and inappropriate impact on clinical care decision-making and behaviors of clinicians because of patient satisfaction score pressures [15,20,21]. At a superficial level, patient satisfaction scores reflect the manipulatable elements of what patients perceive as satisfactory experiences in defined episodes of care, such as hospital stays. In many cases, this indicates environmental factors such as noise levels. Consequently, there is no consistent agreement on whether this is an indicator of the quality of health care delivered or received [8,9,22-24].

### The Integrated Design of Patient Experience and Satisfaction

Understanding how to optimize care delivery requires that both experience and satisfaction be addressed simultaneously. Although there will always be a need to separately understand what has been delivered (patient experience–focused measures) alongside how patients experienced that delivery (patient satisfaction–focused measures) when it comes to designing the environment in which a patient would best thrive in; however, both elements need to be considered. The notion of creating an environment for a patient that is supportive of the broader idea of patient health and well-being, balancing both experience and satisfaction, is an important consequence of using a salutogenic approach and its concept of SOC [25]. Indeed, in response to the conclusions of Dietscher et al [26], this research focuses on the impact of a more patient-centric approach, using IT to improve hospital processes and functioning. It is this broader characterization of experience, as viewed through SOC, which forms the ongoing definition of experience in this paper.

### Experience and the SOC Concept

An approach to this broader concept of experience (ie, patient interactions and responses) is to look at concepts that consider how individuals respond to their environment and how they affect their well-being. One such concept is that of *SOC*, which is a constituent of the Antonovsky Salutogenic Model of wellness [25]. The Antonovsky model is based on an understanding of how an individual responds to stress and the coping mechanisms that the individual has, which enables them to better cope with this stress [27]. “The sense of coherence reflects a person’s view of life and capacity to respond to stressful situations. It is a global orientation to view life as structured, manageable, and meaningful” [28].

The assumption in using this approach is that a reduction in environmental stressors for an individual patient is core to a favorable personal health care experience. Indeed, research on the application of salutogenic orientation in hospitals identifies that interventions improving the hospital design and processes can have an impact on physical health [26]. This is further supported by evidence from psychoneuroimmunology research linking stress and physical health [29]. Although this may not constitute a complete or perfect definition of the drivers of health care experiences, it has proven to be helpful in the care of older adults and health promotion environments where the Salutogenic Model and its concept of an individual’s *SOC* have guided interventions for several decades [30,31].

The SOC core concept in the Antonovsky Salutogenic Model proposes that an individual’s ability to cope with environmental factors that could lead to stress depends on the individual’s three characteristics: their perception of the manageability, comprehensibility, and meaningfulness of their environment, as described in the following sections [32]. Correspondingly, an environment can be optimized in terms of how it contributes to an individual’s well-being (SOC) by optimizing the environment’s ability to deliver the following:

- Manageability: the experience of managing day-to-day physical realities; staying warm, dry, clean, rested, and nourished—the behavioral or instrumental component
- Comprehensibility: the experience of making sense of a situation and creating a structure from otherwise disordered and unexpected information—the cognitive component
- Meaningfulness: the emotional meaning of life and willingness to resolve setbacks and address potential causes of stress—the motivational component

An individual’s SOC can be assessed using several survey-based tools and is an established method of determining an individual’s well-being [33]. The environment can be characterized in terms of generalized resistance resources (GRRs) and specific resistance resources (SRRs) using the Salutogenic Model [34]. Both GRRs and SRRs assist in managing, reducing, or avoiding stressors [35]:

- GRRs are characteristics of a person, group, or community that facilitate an individual’s ability to cope effectively with stressors (tension) and contribute to the development of an individual’s SOC [36]. The social determinants of health and cultural, social, and environmental conditions, such as education, living conditions, salary, self-esteem, and neighborhood, are examples of GRRs [37]. GRRs are less sensitive to direct influence through the manipulation of local technology and information infrastructure.
- SRRs are situation specific and can be optimized to reduce stress in a particular environment or situation; for instance, the ability to change the temperature of a room, use support services, or provide supportive environments. SRRs are elements of experience that can be highly sensitive to direct influences through local technology and information infrastructure.

There is no literature on SRR manipulation to support patients’ experiences. However, SOC has been studied as an overarching philosophy in areas such as nursing [38]. In addition, no literature describes how to model SRRs across an organization to influence a whole hospital population rather than specific patients or in response to postepisode patient experience survey feedback. However, the concept of SRRs has the potential to provide a useful construct for relating the physical and social artifacts within a hospital’s operating environment to their likely impact on the reduction of patient stressors and a change in the SOC-defined experience.

The methodology for this characterization of SRRs in health care, particularly the role of IT in SRRs, is a core component of the intended framework. For information infrastructure design, this work is centered on the assessment of the information infrastructure of a facility in terms of capabilities and our ability to characterize them in terms of their technology composition and their contribution to process outcomes, which in turn generate patient experience.

### Information Infrastructure and Its Assessment

Previous research by Williams et al [3] investigated how information infrastructure can align with health care operational processes. This study resulted in the Infrastructure Maturity Assessment (IMA) framework that enables digital hospitals to assess the maturity of their information infrastructure against their desired digital transformation. The framework characterizes a hospital’s infrastructure maturity to create a road map for digital transformation aligned to operational requirements while simultaneously identifying weaknesses in information and communications technology infrastructure capability. This framework is now an international benchmark of hospital infrastructure performance adopted by Healthcare Information and Management Systems Society Analytics [39]. In this study, we refer to the individual technical capabilities of the IMA process as technology services.

Previous research into the link between patient experience and technology has largely focused on assessing the *soft* or indirect benefits of technology and is primarily concerned with organizational processes [40-42]. Although the IMA framework has, to date, been used to assess the technological competency of a health care facility for supporting their key operational processes, our application here is to use this framework to define an organization’s ability to support the collection of specific process sets that generate desired experiences, as defined through the SOC concept. Hence, the research question addressed is how the patient experience can be supported and improved through the better use of information infrastructure using SOC as a lens to understand the critical areas of technology-responsive improvement.

## Methods

### Overview

This research creates a generalizable framework for improving the patient experience through the better use of information infrastructure, which can be implemented in practice. This framework uses existing knowledge to solve the problem of enhancing patient experience underpinned by theoretical learning. This research is positioned in the applied research discipline of information systems (comprising systems, people, and processes). Design science is the chosen research paradigm as it facilitates the construction of problem solving of real-world challenges, applying theory from other disciplines rather than merely exploring, describing, and making sense of the problem [43,44]. This enables the application of multiple theoretical models to be integrated into design science decisions. The resulting framework (artifact) was developed from, and can be applied to, real-world problems in modeling patient experience.

### Research Design

**Figure 1:**
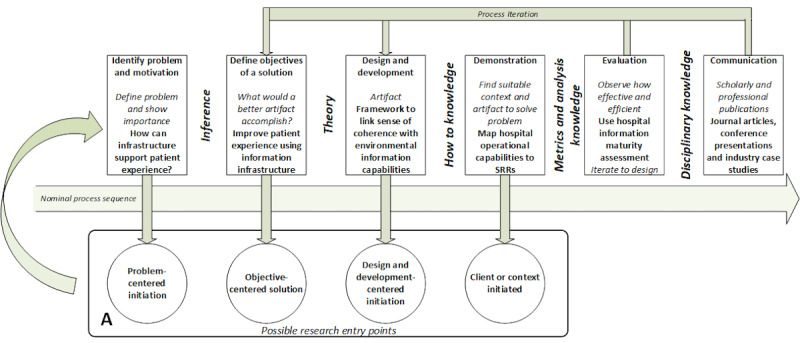
Research design based on the Design Science Research Methodology [39]. SRR: specific resistance resource.

The research design defines the contextualization of the methodology for the research question. The application of the Design Science Research Methodology (DSRM) in this research is shown in Figure 1. Each DSRM activity is explained along with its corresponding output in the *Results* section.

## Results

The results reflect the steps of each DSRM activity in the research design, as shown in Figure 1.

### DSRM Activity 1: Problem Definition

This study had a problem-centered initiation entry point (label A in Figure 1). The underlying problem in many approaches to patient experience is that they hinge on assessing experience using postepisode surveys, targeting only specific processes for improvement. Postepisode surveys generate little insight into what drives individual patients to form their experiences and what is an optimal experience (a set of interactions and responses) for a patient. The survey approach, although critical in assessment, is insufficient for the purposeful design of patient environments. Experience-based design requires a more holistic model that balances individual patient preferences and benefits. Currently, there is no method for modeling how to improve the patient experience by examining the role of information infrastructure and services in the delivery and support of patient experience. Such a model needs to generate insight into the underlying drivers of such a balanced positive outcome and what influences these drivers. In this study, it was imperative to have those drivers defined in terms relevant to the impact of information infrastructure.

### DSRM Activity 2: Solution Objectives

#### Overview

**Figure 2:**
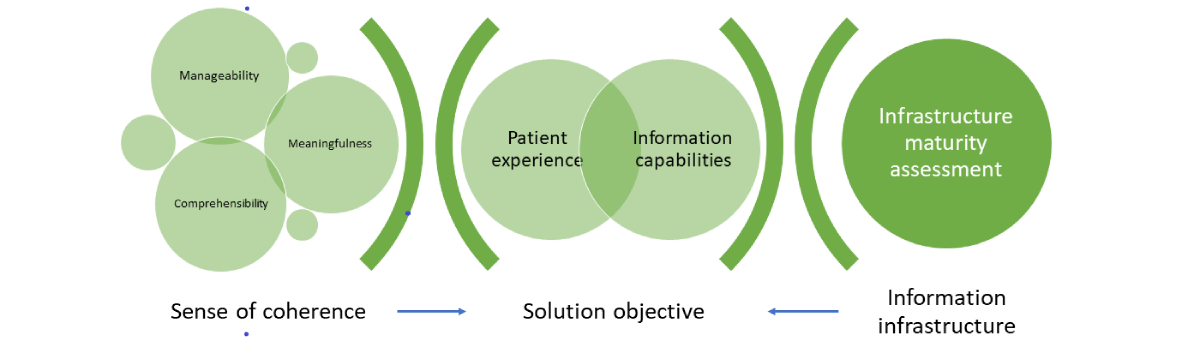
Research design: Design Science Research Methodology activity 2—forming the solution objective.

The solution’s objective was to improve the patient experience by reducing environmental stressors for a patient (physical, mental, and social) through the existing hospital information infrastructure. Using the SOC concept as the perspective through which to support care provision in the hospital environment provides a method for defining what a positive and supportive experience looks like and linking this to the information capabilities of the hospital’s operational environment. This activity was performed from two sides: defining existing or aspirational patient experiences within the health care environment at one end using SOC and, from the other end, defining how technology comes together to form processes using the IMA framework (Figure 2) that supports those experiences.

The separate actions in this activity (as shown in Figure 2) were as follows:

1. Mapping the hospital patient experience in terms of the SOC concept, detailing the experience statements that describe the domains of manageability, comprehensibility, and meaningfulness in terms of the operational environments of people, places, and processes
2. Describing how technologies and their combined technological capabilities come together to form information capabilities accessed through the IMA framework
3. Describing how information capabilities combine to form the SRRs that support experience statements.

#### Mapping the Hospital Patient Experience in Terms of the SOC Concept

The first action involved mapping the hospital patient experience in terms of the SOC concept, detailing the experience statements that describe the domains of manageability, comprehensibility, and meaningfulness in terms of the operational environments of people, places, and processes.

SOC is defined through its three domains: manageability, comprehensibility, and meaningfulness. It is helpful to view these domains in the context of the three environments in which patient experience is often discussed: the operational environments of people, places, and processes. This is the starting point for mapping the experience requirements of a care organization. It is the point at which the organization’s vision, mission, and values are distilled into a set of statements that describe the organization, either as it exists at present or as it aspires to be in the future (Table 1).

Each SOC domain can then be articulated relative to these environmental descriptions using a set of experience statements (Textbox 1). These experience statements are not absolute descriptions of the ultimate state of an organization (although, in time, these types of descriptions could evolve); rather, they are distilled from the mission, vision, and values of the organization they describe. In Textbox 1, we articulate a typical set of statements for a modern acute care facility. We have structured the statements to include the condition of the environment (where relevant) and the desired experience, as the condition is an enabler (not a cause) of the subsequent experience. The experience statements are for an individual care organization based on the already defined operational environment descriptions. These statements are defined in collaboration with the facility’s clinical and operational staff and their patients and represent either the organization’s current state or the future aspirational state of the organization. They are defined for the three operational environments: people, places, and processes.

**Table 1:** Sample operational environment experience statements for an acute health care organization.

Statements and examples	Place	People	Process
Operational environment experience statements	“I can individualize my external environment in such a way that best supports my needs.” “The environment is such that there are a minimum number of distractions.” “The environment is responsive to my emotional state and creates a calm and supportive atmosphere.” “The environment can be easily customized to my specific needs, and it reliably stays that way. It produces environmental changes that are traceable, and the logic is transparent.”	“My care team respects my preferences, beliefs, and values, and I have jointly agreed to the goals of care that I can influence in an ongoing way.” “I find working with my carers enriches my life and expands my goals for myself.” “I feel listened to and valued by my carers. There is clear communication between my care team that enables me to feel the I have control.” “There is a close and reliable bond established with the care providers that work with me.”	“I can engage with and appropriately manage the processes and systems to support me in a manner that is optimized to my preferences.” “There is effective coordination between the care team members. There is continuity of care with smooth transitions from one setting to another.” “Processes are responsive to my emotional state and are flexible.” “I feel that the processes are reliable and effective.”
Examples	Temperature, humidity, luminosity, color (hue, saturation, value, and color temperature), noise level, tactile suitability, navigability, cleanliness, enjoyability, comfort, and connectivity	Friendliness, hospitality, teamwork, cooperation, rapport, transparency, responsiveness, sensitivity, empathy, truthfulness, behavior, professional etiquette, competency (cultural, spiritual, and clinical), accountability, awareness, capability, mastery of the systems (social and technical), respect, and communication	Interoperability, completeness, reliability, availability, security, resilience, agile, adaptable, simplicity, patient centric, effective, efficient, optimized, empathetic (accommodating to individual circumstance and personalization), well-defined, understandable, engaging (includes user experience), sustainable, acceptable, ethical, legal, fair, equitable, reasonable, coordinated, integrated, safe, and timely

**Sample of typical sense of coherence experience statements.**

**Environment: place**

- “I can individualize my external environment in such a way that best supports my needs.”
- “The environment is such that there are a minimum number of distractions.”
- “The environment is responsive to my emotional state and creates a calm and supportive atmosphere.”
- “The environment can be easily customized to my specific needs, and it reliably stays that way. It produces environmental changes that are traceable, and the logic is transparent.”

**Manageability**

- “I can influence or control the environment.”
- “I have sufficient information about the healthcare environment to form reasonable expectations.”
- “The environment provides sufficient amenities and facilities to reduce stress and enhance well-being. I feel more able to be in a positive mood due to an environment tailored to my personal preferences.”
- “The environment is designed in a way that builds reliability. My environmental needs will be taken seriously. The environment is uniform and consistent with my specified requirements. It is responsive to my needs.”

**Comprehensibility**

- “I understand what I can influence within the environment.”
- “The information I have on the healthcare environment is presented in such a way that it is understandable by me (plain language, translated, visual and text)”
- “The absence of excessive environmental demands (noise, crowding, clutter, unclear signage, accessibility) enables a better understanding of the information provided to me.”
- “I can direct my attention and focus on what is relevant. The environment reduces the simultaneous demands and minimizes distractions.”

**Meaningfulness**

- “I can exercise my personal preference to build my capacity to make choices about my health now and in the future.”
- “Having choices of the environment reinforces my belief in being able to influence my future positively.”
- “I feel safe. The environment creates the context for who I am and what I have done. It allows me to interact easily with others creating a greater sense of belonging.”
- “The environment supports my exploration of meaning-making by reducing fatigue and stressful demands. It encouraged and supported investigation beyond maintaining daily function. My ability to reliably engage with relevant information sources and share information between key people engenders a high level of trust in the environment.”

#### Describing How Technologies and Their Combined Technological Capabilities Come Together to Form Information Capabilities Using the IMA Framework

A multistage process was used to describe how information capabilities can be formed. The first stage was formed around the IMA, which aggregates technologies into technology services (across the five domains of technical service capabilities of the data center, security, collaboration, mobility, and transport) and structures those services according to a staged maturity matrix [3].

In the second stage, the technical service capabilities of the domain are linked to form *information capabilities* (Textbox 2). Information capabilities are characteristics that information systems require for data actions in end user services. Information capabilities come together to support and create the processes across the three operational environments:

- People (or resources) using the infrastructure (eg, administrators, patients, staff, and equipment)
- Places where the information systems are used (whole hospital, specific hospital units, externally dependent campuses, and car parking)
- Processes that are dependent on information systems (nurse calls, bed management, and task management)

Textbox 2 describes the information capabilities within the operational environments of people, place, and process. Each information capability is defined in terms of the technical capabilities from which it is constructed. The assignment of technical capabilities to an individual’s information capability is dependent on the real or aspirational operational objectives of a health care organization. The assignments described in this paper were allocated according to the operational objectives defined for an advanced digital hospital operating at level 8 of the IMA.

**Information capabilities descriptions.**

**Place**

- Accessing: to establish interaction with resources (eg, people, equipment, supplies, information, and systems)
- Controlling: to influence resources (eg, people, equipment, supplies, information, and systems)
- Alarming: to notify the occurrence of a negative (problematic) event
- Alerting: to notify the occurrence of an event
- Measuring: to quantify the characteristics of a resource (eg, people, equipment, supplies, information, and systems)
- Responding: to create an action in response to an event

**People**

- Sharing: the exchange of data (including textual, image, or graphical information); it can be both synchronous and asynchronous; restricted to permanent and semipermanent file types (retrievable data types)
- Communicating: remote voice and video conversations between individuals or groups; face-to-face gathering of people; the synchronous or asynchronous exchange of textual information in a threaded and persistent form
- Locating: being able to identify how to access resources (eg, people, equipment, supplies, and information) in places
- Recording: the transcription of transient voice and visual information into a permanent record
- Organizing: arranging the schedules of one or a group of people and resources
- Identifying: describing the characteristics of people, places, or things in sufficient detail to uniquely define them
- Analyzing: the processing of information to form insights into decision-making
- Requesting: the identification of a need for a person, place, thing, or process so that it can be supplied at a given time or place
- Interpreting: analysis of current situations or information

**Process**

- Interoperating: the ability for processes to interact in a way that generates the desired outcome
- Contextualizing: creating information or processes that are relative to an individual’s characteristics and the characteristics of the environment around them
- Orchestrating: scheduling, timing, and location of resources to maximize outcomes
- Scheduling: establishing the timing and location of services
- Simplifying: reducing complexity
- Informing: to make people or systems aware of relevant events or information
- Tasking: to assign a specific activity to an individual, group, or process
- Trusting: the creation of secure systems in which information is shared only within the rules established by the organization

Technical capabilities may be considered common (pertaining to all information capabilities within an operational environment) or specific (related to ≥1 but not all information capabilities within an operational environment). In summary, infrastructure-related technologies are used to create technical capabilities, and the aggregation of these technical capabilities forms information capabilities. Using this methodology, we can define an organization in terms of the maturity of its technical capabilities and in terms of the maturity of its information capabilities.

Information capabilities were graded using a modified (4-step) version of the 8-step IMA assessment. The IMA relates to how technology affects processes and is supported by a large volume of data. The 4-step maturity scale in Table 2 reflects a summary of the IMA 8 steps because of the current limited understanding of how hospitals mature in their delivery of the experience. In the future, it may be possible that this scale is expanded as more data are available and more granularity in defining the stages to improve patient experience in hospitals is gained. At this stage, only 4 steps could be assigned with confidence.

The 4-step information capability scale is described in terms of typical outcomes for each level across the operational environments of place, people, and processes (Table 2). This assessment provides organizations with an understanding of the technical strengths and weaknesses of the major operational environments.

**Table 2:** Information capability maturity—a 4-step maturity scale is used to assess information capability maturity within a health care facility.

Level	Place	People	Process
Level 0: fragmented	Data about the environment, the patient, and the staff may not be accurate or comprehensive because of infrastructure challenges and information capability issues. The format may be understandable but cannot be accessed easily.	It may not be possible for us to share clinical, environmental, and operational information between relevant individuals and groups. Without sharing, we may not be able to add to and refine this knowledge or develop a course of action to achieve our objectives.	An individual or group may not be able to take the plan of action and implement it by delivering physical resources, people, and knowledge to the appropriate places and locations within the organization at the required time. The actions of individuals linked with other individuals and teams coordinated with the assistance of the operational systems within the facility may be compromised.
Level 1: informed	Data about the environment, the patient, and the staff are accurate and comprehensive. It is accessible easily in an understandable format.	One can share clinical, environmental, and operational information between relevant individuals and groups. We can add to and refine this knowledge, developing a course of action to achieve our objectives.	An individual or group can take the plan of action and implement it by delivering physical resources, people, and knowledge to the appropriate places and locations within the organization at the required time. This would encompass the actions of individuals linked with other individuals and teams coordinated with the assistance of the operational systems within the facility.
Level 2: cooperative	Information is in a format and on a system that one feels comfortable using and has sufficient skills to operate effectively. The information is in a language that one is familiar with. One can interpret its content and purpose and gain further insight into the specific situation related to him or her and the course of action that needs to be pursued.	One feels closely connected with their care team, family, and social networks involved with his or her recovery. They understand his or her situation and the ways that they can best support him or her. They feel connected and invested with their situation and action plan. They can seamlessly share information and build collaborative plans to support their objectives.	The operations of relevant systems for delivering one’s care are accessible, transparent, and understandable to their care providers and them. They are presented in a way that one can optimize their application for his or her specific outcomes (within the constraints of optimizing the whole of system outputs).
Level 3: systemized	The information is relevant to one’s individual needs and future aspirations. The information enables one to cope with his or her daily challenges more effectively, providing a more effective sense of control of his or her outcomes. It allows him or her to craft an understanding of their future that is hopeful yet respectful of challenges that one will face in achieving that future.	Individuals can readily share the information with others to enable them to gain further understanding of their situation and course of action. One can build closer and more supportive relationships with members of his or her team (either patient or clinical) and feel an increased sense of engagement and control because of this.	One feels in control of their care. They understand all the resources at their disposal for optimizing the path to their future objectives. One feels that one has control over those resources, and they coordinate with each other to minimize their intervention in their delivery. They are linked with their care delivery team, and they evolve the services they deliver and how those services are provided, dependent on their progress to recovery.

#### Describing How the Information Capabilities Combine to Form the SRRs That Support the Experience Statements

The four main classes of information-driven SRRs within a hospital were defined through clinical and operational interviews as follows: teaming and sharing, scheduling and coordinating, monitoring and reporting, and education and training.

Information capabilities are rated according to their relevance to an SRR class. In analyzing an existing facility, the major applications and processes that constitute the SRR classes are defined, and the relevance of the facility’s information capabilities is estimated using a 4-step scale (Table 2). This provides a map of SRR classes and their information capability strengths. The relevance of SRRs to the experience statements within the SOC domains (manageability, comprehensibility, and meaningfulness) can then be established using an equivalent relevancy scale. The process of estimating the relevance of both information capabilities to SRRs and SRRs to SOC domains is a critical part of the modeling process that engages a hospital in understanding the information capabilities they have and how they could, or do, affect the patient experience.

### DSRM Activity 3: Design and Development

#### Overview

The design and development activity details the process used and results for each step in the framework creation, leading to the final link between the information infrastructure and patient experience. The solution objective was designed by refining the two ends of the solution (information infrastructure and SOC) into common SRRs that describe both the experiences to be delivered and the technological competency to deliver those experiences.

The experience requirements and technology contributions to the SRRs were described in terms of both their operational environments (people, places, and processes) and the SOC domains (manageability, comprehensibility, and meaningfulness) using the DSRM 2 outputs of experience (Textbox 1) and IMA-based information capability outputs.

#### SRR Development

When combined and applied by people in a health care organization, information capabilities result in information-based SRRs and are the aggregation of people with technology to generate processes. The four major classes of information-based SRRs are teaming and sharing, scheduling and coordination, education and training, and monitoring and reporting, as described in Textbox 3. These classes were developed based on an experience study conducted at Fiona Stanley Hospital in Perth on their Enhanced Recovery After Surgery service and were established through extensive discussions with clinicians, technologists, and health care providers.

Through our earlier analysis, we defined the information capabilities that were then quantified through the extension of the IMA process across operational environments (people, places, and processes). In addition, we have experience statements across the operational environments (people, places, and processes) defined at the level of the SOC domains (manageability, comprehensibility, and meaningfulness). It is now possible to link these 2 sets of data together by building specific experience statements for each level of information capability assessment (fragmented to systematized) across each of the SRRs at the operational environment level (Table 3 and Textbox 4). This allows an organization to rank the relevance of its information capabilities to the SRRs they consider most relevant to the type of care they wish to deliver. Table 3 and Textbox 4 show a sample of the process-relevant experience statements for each level of information capability assessment for each of the SRR categories. Equivalent capability level experience statements are created for the information domains of people and place.

**Figure 3:**
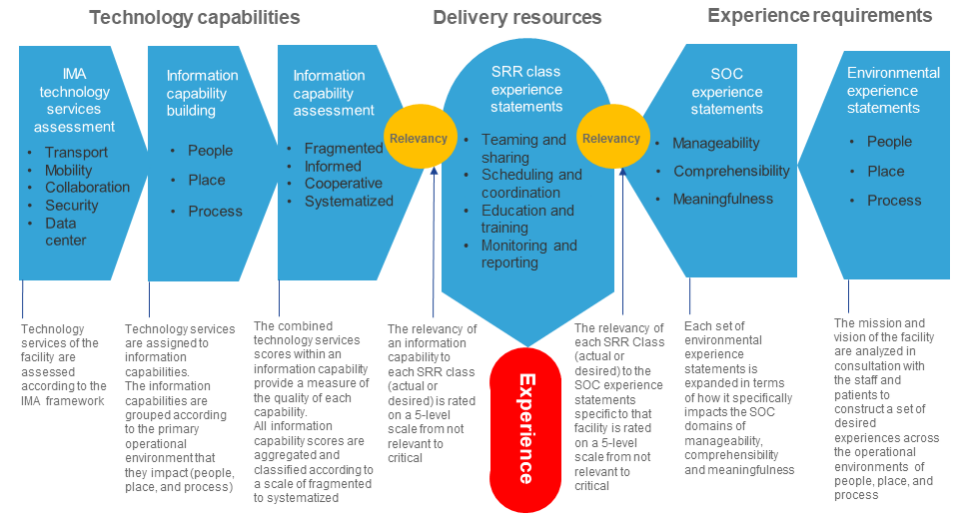
Process flow for linking experience requirements with technology capabilities to enable the delivery of required SRRs. IMA: Infrastructure Maturity Assessment; SOC: sense of coherence; SRR: specific resistance resource.

An overview of how the technological capabilities and experience requirements were drawn together through the creation of SRRs is depicted in Figure 3. SRRs are described both in terms of their technological components and their inherent experience statements and consequently form a bridge between technology and experience. This forms an overarching *Information Infrastructure to Experience Framework* (presented in the *Discussion* section, together with a discourse on how the framework may be used).

**Figure 4:**
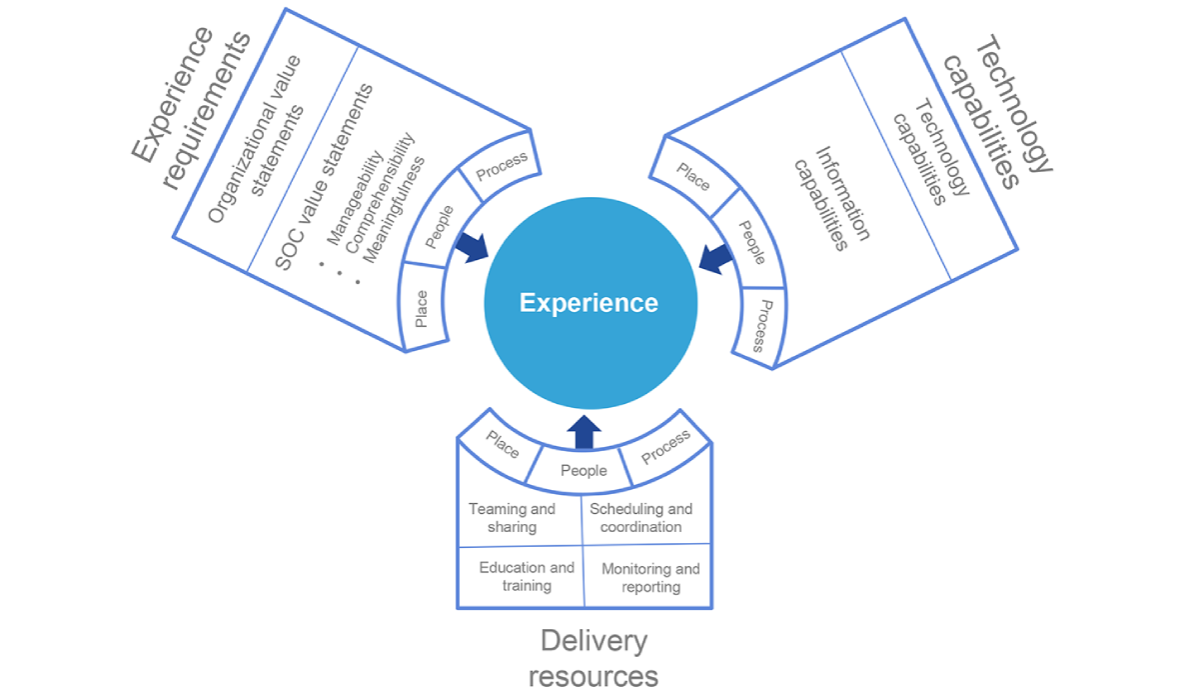
Information infrastructure to experience framework. SOC: sense of coherence.

The process flow depicted in Figure 3 can be simplified to the high-level framework description shown in Figure 4. This Information Infrastructure to Experience Framework draws together the three key characteristics of the information infrastructure–driven experience landscape: technology capabilities, experience requirements, and delivery resources. It emphasizes the critical requirement of describing each of these elements within the common operational environments of people, places, and processes. Through this process, it is possible to relate the information infrastructure requirements to support the delivery resources needed to achieve the desired experience.

**Definitions of specific resistance resources.**

**Teaming and sharing**

- Simply and conveniently bringing together clinicians, patients, and carers in the most appropriate format (pairs, groups, teams, and embedded into clinical workflows) to share information and emotion and enable the processes of care delivery and social support, minimizing the barriers of distance and timing

**Scheduling and coordinating**

- Linking clinical, patient, and carer engagement with scheduling and booking functions within the hospital to enable clear communication and management of activity timing to all participants, staff, and systems in each stage of an individual’s patient journey

**Monitoring and reporting**

- The ability of patients, carers, and clinicians to access, interpret, and add to patient progress data; evaluate patient compliance; and modify the engagement to optimize the clinical and personal outcomes

**Education and training**

- The provision of education, training, and research materials at the appropriate time and appropriate format, which best supports the patients’ clinical and personal needs and the clinician’s requirements for decision-making and development

**Table 3:** An extract of the operational environment characteristics of experience (fragmented to systemized) for each specific resistance resource.

Specific resistance resource	Operational environment information capability maturity level	
	Information capability level 0: fragmented^a^	Information capability level 3: systemization^b^
Teaming and sharing of information contribution	Task assignment and status are somewhat articulated and are not readily accessible to the individual. Process structure and status are articulated but may not be readily accessible to the individual. The skill sets and availability of individuals to accept tasks are articulated but may not be readily available to the individual. An individual’s workload is not readily accessible.	Individuals have access to technologies that enable them to optimize the allocation of tasks so that they best fit the skill sets, work demands, work environments, and available technologies of the individual to whom the task is assigned. Individuals have access to the technologies that enable them to define, allocate, and form tasks set into overall processes that sequence around the needs of the individual and the resources that are available within the organization.
Scheduling and coordination of information contribution	The interactions between component tasks and the overall processes they drive may not be clearly defined and not readily accessible to the individual.	Individuals and teams can conveniently coordinate tasks, managing those assigned and their sequencing (both in time and with respect to other necessary precursor events).
Education and training of information contribution	The training and education activities do not articulate the processes that combine to create the required care delivery and how the component activities create the desired outcomes.	The training and education process enables the individual to understand how to customize their educational resources to their current and predicted future needs, both personal and professional. They enable Individuals to choreograph their education and training programs around an existing potential future commitment.
Monitoring and reporting of information contribution	How processes deliver upon supporting an individual’s culture and values may be monitored and reported on and may not be accessible to all relevant personnel. How current processes interact to support the quality and reliability of an individual’s support services are not regularly monitored and reported on and may not be accessible to all relevant personnel. How processes deliver upon supporting an individual’s culture and values is monitored and reported on to be accessible to all relevant personnel. How current processes interact to support the quality and reliability of an individual’s support services is regularly monitored and reported on so that it is accessible to all relevant personnel.	The efficiency of processes working in isolation or in more complex systems is monitored and reported, particularly looking to reduce complexity and potential bottlenecks in process execution.

^a^Information capability domain score average: 0.00-0.90; data about the environment, the patient, and the staff may not be accurate or comprehensive because of infrastructure challenges and information capability issues. The format may be understandable but cannot be accessed easily.

^b^Information capability domain score average: 2.41-3.00; an individual or group can take the plan of action and implement it through the delivery of physical resources, people, and knowledge to the appropriate places and locations within the organization at the required time. This would encompass the actions of individuals linked with other individuals and teams coordinated with the assistance of the operational systems within the facility.

**An extract of the operational environment characteristics of experience (experience statements for processes) for each specific resistance resource.**

**Manageability**

- “I can tailor aspects of my care within the larger process of a health organization.”
- “The process demands are reasonable and allow for choices and the needs of my life outside the health organization.”
- “Tension is reduced because the process is efficient and effective and conforms to my evolving needs.”
- “The processes are knowable, reliable, and effective, and I have developed confidence in them.”

**Comprehensibility**

- “I understand my rights and responsibilities within the processes of health.“
- “The processes are knowable and predictable.”
- “Processes are clearly explained, and I understand my role, and when something is not right, I can voice my concerns and those concerns are heard and responded to”
- “The process is understandable, fair, and equitable for me.”

**Meaningfulness**

- “The choices I make, and the choices offered to me align with my care goals and desired health outcomes.”
- “I see the processes as parts that form a whole. They move me closer to my end goal.”
- “Despite the volume of processes, I see them culminating in value for my treatment and care goals.”
- “I believe that the process aligns with the goals of care and the outcomes I seek.”

### DSRM Activity 4: Demonstration

Through the combination of information capabilities into applications and processes, grouped together as SRR classes (collectively termed delivery resources), the linkage of technology capabilities with experience requirements was assessed to determine how the framework can be applied using existing IMA data. The assessment provided a proof of concept that demonstrated the use of the framework in practice.

Subsequently, there are 2 ways to demonstrate the Information Infrastructure to Experience Framework (Figure 4). First, it can be used in a descriptive fashion to explore the experience requirements (experience landscape) and possible technological responses to the landscape. This is addressed further in the *Discussion* section, along with the potential impact of this approach.

Second, it can be used in an analytical fashion to directly assess the current digital infrastructure’s capability to support the organization’s experience goals. In this process, an organization’s information capabilities are scored using a modified IMA process. The relevance of these information capabilities to the organization’s experience landscape is then rated by defining and analyzing the relevance of the organization’s SRRs.

The first step of the analytical process comprises defining the information landscape by establishing sets of experience statements at the following levels:

- Operational environment (people, places, and processes)
- SOC domains (manageability, comprehensibility, and meaningfulness)
- Major SRR classes (teaming and sharing, scheduling and coordinating, monitoring and reporting, and education and training)

This defines the experience requirements of the organization as described in Table 3 and Textbox 4.

The second step is the analysis of the information capabilities. The information capabilities, as established for each of the operational environments (people, places, and processes) in Textbox 2, are assigned applicable technology services. These are the same technology services as described in the IMA research [3]; however, these technology services are assessed on a 4-level maturity scale (Table 2) in contrast to the 8-level IMA maturity scale. This generates an assessment of the technological service contribution to each information capability (see the sample in Table 4). Table 4 shows an extract from a much larger matrix that details the technological service requirements to reach a given experience performance level within the operational environments of people, places, and processes. Table 4 focuses on a selection of the IMA transport domain technological capabilities and how they are accessed in the operational environment of place.

Each information capability was then ranked according to its relevance to each of the 4 sets of SRRs, using the relevancy levels described in Textbox 5. The relevancy scale is used to define the level of importance of an information capability to an SRR, thereby indicating its significance in delivering the SRR. A simple 5-level ranking scale was selected as a smaller scale would be insufficiently definitive, and a larger diversity in rank would potentially create unnecessary differentiation in relevancy and would not add value to the relevancy assignment task.

**Table 4:** Scoring criteria of the Infrastructure Maturity Assessment technological capabilities on the 4-step experience scale.

Technological capabilities	Level 0	Level 1	Level 2	Level 3
Virtualization	Virtual segmentation of campus infrastructure is based on static configuration.	Macro–virtual segmentation of campus infrastructure is based on VLAN^a^ trunking protocol propagation and VRF^b^.	Micro–virtual segmentation of campus infrastructure is based on VxLAN^c^.	Access controlled, policy-based microsegmentation of campus infrastructure is based on VxLAN.
End of support status	End of support status applies to ≤5% of core and distribution layer technologies and ≤30% of access layer technologies.	End of support status applies to ≤5% of core and distribution layer technologies and ≤20% of access layer technologies.	End of support status applies to ≤3% of core and distribution layer technologies and ≤10% of access layer technologies.	End of support Status applies to ≤3% of core, distribution, and access layer technologies.
Wired device grade	Approximately ≤70% of switches and routers are enterprise grade.	N/A^d^	Approximately 71% to 97% of switches and routers are enterprise grade.	Approximately >98% of switches and routers are enterprise grade.
QoS^e^	Fragmented QoS within the health care entity campus has been implemented. Trust boundaries are well defined.	End-to-end QoS has been implemented within the health care entity campus. Trust boundaries are well defined.	End-to-end QoS has been implemented within the health care entity campus and across the WAN^f^. Trust boundaries are well defined.	SDN^g^ controllers have been implemented and are used to provide business applications and dynamic end-to-end QoS within the health care entity campus and across the WAN. Trust boundaries are well defined.

^a^VLAN: virtual local area network.

^b^VRF: virtual routing and forwarding.

^c^VxLAN: virtual extensible local area network.

^d^N/A: not applicable.

^e^QoS: quality of service.

^f^WAN: wide area network.

^g^SDN: software-defined networking.

**Relevance of an information capability relevancy to a specific resistance resource.**

**Rank 1**

- Not relevant or rarely involved; the specific resistance resource is not required or provides information contribution based on safety requirements

**Rank 2**

- Occasionally involved; the specific resistance resource provides information contribution based on safety and timeliness requirements

**Rank 3**

- Normally involved; the specific resistance resource provides information contribution based on safety, timeliness, and efficiency requirements

**Rank 4**

- Should always be involved; the specific resistance resource provides information contribution based on safety, timeliness, efficiency, and effectiveness requirements

**Rank 5**

- Critical, must always be involved; the specific resistance resource provides information requirements based on safety, timeliness, efficiency, effectiveness, equity, and sustainability requirements

The third step establishes the relevance of the SRR classes to the organization’s overall SOC-defined experiences within the domains of manageability, comprehensibility, and meaningfulness. This characterizes an organization’s technological capability to deliver on the desired SOC experiences. In this process, each of the SRR classes is rated with respect to their relevance to each of the SOC domains (as defined by their experience statements) using the 5-point scale used in Textbox 5. Rating the SRR’s technology capability by its relevance to an SOC domain generates an overall capability score (SOC domain-weighted experience capability) for each of these domains. A sample of this process is presented in the following section*, DSRM Activity 5: Evaluation*.

### DSRM Activity 5: Evaluation

#### Overview

The preliminary evaluation used existing Australian data from past hospital infrastructure maturity assessments to ensure that the framework was robust yet flexible when applied to different health care environments. This research had two major outputs: the Information Infrastructure to Experience Framework and the framework scoring.

The initial evaluation tested the process by observing how the model responded to data inputs from the existing IMA data and taking those outcomes through the SOC experience statements. The next step in the evaluation was to perform detailed assessments using a framework with specific hospitals. For this, the first requirement was to define each of the core components of the experience framework across the operational environments of people, places, and processes.

#### Experience Requirements

This involves the outcome that the organization seeks to deliver through its experiences, described in terms of the patient experience statements for the SOC domains of manageability, comprehensibility, and meaningfulness.

#### Technology Capabilities

The information infrastructure maturity of the organization derived from the IMA quantifies the maturity of the technology services that are assembled into the information capabilities of the organization.

#### Delivery Resources

This involves the information-based processes within an organization that can use the information capabilities to deliver patient experience statements. These information processes form the SRRs defined within the concept of SOC.

The second step is ranking the information capabilities in terms of their relevance to the SRR classes and then ranking the SRR classes in terms of their relevance to the experience statements that define the three SOC domains of manageability, comprehensibility, and meaningfulness.

#### Framework Scoring

A preliminary evaluation of the information capability scoring and its relevance to the SRR classes is provided as a worked example in Multimedia Appendix 1. This example is based on a previous IMA conducted on an Australian hospital. The framework scoring (Multimedia Appendix 1) demonstrates the competency of a facility’s information infrastructure to support the technology requirements of a given set of SRRs for each of the operational environments of people, places, and processes. The framework scoring process considers the relevancy of each SRR to each of the SOC domains, as defined by an individual hospital’s experience statements, to generate a final SOC domain-weighted experience capability based on their current technology infrastructure.

With an understanding of the technological strength of an organization’s SRRs, the final issue is understanding the relevance of SRRs to achieving the experiences the organizations aspire to deliver. This study provides a link between technology and experience. This could be achieved by taking the experience statements derived through discussion and analysis with the organization’s clinical staff, operational staff, and patients and ranking the relevance of the SRR groups. The relevance of each SRR to support the desired experience statements within the SOC domains of manageability, comprehensibility, and meaningfulness was estimated using the relevancy ranking in Textbox 5. The framework score reflects the competency of the information infrastructure to support a given level of experience within each SOC domain of manageability, comprehensibility, and meaningfulness.

#### Future Evaluation

The next phase of the evaluation process will be undertaken from 2022 to 2023 with specific hospitals to validate the framework using expert reviews and, subsequently, implement the framework with a selection of Australian hospitals.

### DSRM Activity 6: Communication

The communication of this research is initially through this paper, detailing the complex research process and the body of work. In addition, an industry case study is in preparation, along with a technical report on the use of the framework for the industry. As the framework is designed for practical use, innovation and usability factors are essential for communication.

## Discussion

### Principal Findings

The developed framework uses the concept of SOC as a lens through which to view and define patient experience in the context of reducing environmental stressors for patients. Through this approach, the framework demonstrates the links among the critical perspectives of experience, supporting information capabilities, and information infrastructure. In addition, the maturity of these supporting capabilities can be measured using a capability maturity assessment model based on the established digital infrastructure assessment methodology [3], and pathways for improvement can be identified.

The purpose of the framework is to assist hospitals in improving their effectiveness regarding patient experience by connecting and orchestrating the synergy among people, processes, and systems using the organization’s infrastructure capability. To this end, the framework can be used in two ways: to contextualize and generate conversation for improvement in patient experience and as an assessment tool to evaluate the current information infrastructure.

### Contextualization

The concept of SOC provides a pragmatic structure for establishing the overall experience objectives within a health care organization. These guiding principles can then be reflected in the desired experiences at the operational environment levels of people, places, and processes. These, in turn, can contextualize the experience requirements for the way in which IT-driven operational processes (SRRs) interact with the patient. Such an experience map of an organization enables a clear definition of the information infrastructure requirements to support these desired experiences.

This approach enables the model to tap into the rich archive of survey-based empirical research to guide the experience statements, which are a critical part of the major stages of this model. This framework can be used as a road map for specific improvements, generating discussions on aspirational experiences and how to reach them. In this way, it assists in the design of patient experience road maps rather than journey maps.

### Assessment

There are 2 aspects of this assessment. The first is evaluation, and the second is the scoring methodology.

It is possible to use the framework to assess the current capabilities against the draft experience statements contained in the framework or to distill the organization’s vision, mission, and objectives into a revised set of customized experience statements. In doing this translation from organizational goals into organization-specific experience statements, it is possible to assess the organization’s ability to meet those experience goals, identify gaps, and establish improvement strategies.

The second aspect is to use a scoring methodology to assess the ability of the current information infrastructure to support the desired experience. To score an organization’s existing information infrastructure capability, an SOC was established along the lines of a balanced scorecard, with manageability, comprehensibility, and meaningfulness assessed independently. The organization’s culture and objectives define the balance of these components. It is particularly insightful to apply the grading detailed in the framework scoring example in Multimedia Appendix 1 at the individual SOC domain level of manageability, comprehensibility, and meaningfulness and reflect it back to the vision of the hospital.

### Summary

The final output of this framework is the capability of an organization’s information infrastructure to support the desired SOC for the organization and, in doing so, create an explicit set of experiences supportive of positive patient outcomes. The innovation of this research is that, traditionally, SRRs are used when tensions are perceived to create stress [34]. However, our research challenges this perception to prevent the underlying issues in the first place rather than wait until they are perceived as threatening. In this way, it models the prevention of potential threats across the cohort in a unique hospital situation. This ensures that the right SRRs, using the information infrastructure, are available when needed and are not left to chance.

### Significance

This research represents a novel approach, which does not currently exist in the literature, in specifying how SRRs can be explicitly designed to support the patient experience. Perhaps, more uniquely, it defines how the facility’s information infrastructure can be designed to best support the role of those SRRs.

This study used the SOC concept to construct a bridge between patient experience process measures and patient satisfaction outcome measures. In doing so, we created an integrated model of how information capabilities using technology can enhance the delivery of care, which has not been done before. The development of the Information Infrastructure to Experience Framework as a process capability framework will assist in the practical application of (service innovation) experience-driven improvement, specifically in supporting capability and collaboration development. This contributes to developing operational capabilities and the assessment or measurement of these operational capabilities.

Finally, regarding the research methodology, using the DSRM with additional embedded theory in the design and development arguably demonstrates a more advanced and complex application of the research paradigm than is typically seen.

### Future Research Opportunities

The question of the amount of information infrastructure that affects the activities of experience in practical implementation is still to be fully investigated. The type and definition of information capabilities, as well as the classes of SRRs, will benefit from ongoing exploration, together with further testing of the weighting processes in a wider variety of health care settings.

The next step in this research is the validation of the experience statements and case studies in the use of the framework. In addition, the investigation into the use of the framework methodology to define experience using an SOC in other sectors that are looking to take a novel approach to improve the experience is in progress. These include universities and subsets of specific clinical contexts such as cancer survivorship and complex drug therapies. Furthermore, although this research has focused on patient experience, it should be acknowledged that there would be an analogous process for staff experience.

### Conclusions

Given the complex nature of experience in health care and to enable the creation of a coherent and simplified framework, we focused our experience definition on the impact of, as well as our ability to manage, environmental stressors. In doing so, we can use the well-established concept of SOC to describe the processes of stressor reduction. We linked this to the established model of information infrastructure service, the IMA, through the concept of information capabilities and the SRRs they support.

Through this approach, this research has demonstrated information infrastructure to experience mapping, taking the theory and characteristics of salutogenic SOC to inform the articulation of a positive patient experience and how this is supported by the information infrastructure. This is defined in both technological and experience terms at the levels of the operational environment (people, places, and processes) and through the delivery resources (SRR classes: teaming and sharing, scheduling and coordinating, monitoring and reporting, and education and training).

## Appendix

Multimedia Appendix 1 Worked example of information capability scoring and its relevance to the specific resistance resources.

## Abbreviations

DSRM: Design Science Research Methodology
GRR: generalized resistance resource
IMA: Infrastructure Maturity Assessment
IT: information technology
RMIT: Royal Melbourne Institute of Technology
SOC: sense of coherence
SRR: specific resistance resource
