# Supplementary material for: A Sense of Coherence Approach to Improving Patient Experience Using Information Infrastructure Modeling: Design Science Research
Source: JMIR Form Res. 2022 Apr 12;6(4):e35418. doi: 10.2196/35418 (PMC9044153; doi:10.2196/35418)
Supplement: Multimedia Appendix 1 [file formative_v6i4e35418_app1.docx]

# Worked example of information capability scoring and its relevancy to the Specific Resistance Resources.

## Appendix 1

Framework Scoring: The preliminary evaluation of the information capability scoring and its relevancy to the Specific Resistance Resource (SRR) classes is provided as a worked example in Table A. This is based on a previous Infrastructure Maturity Assessment (IMA) conducted on an Australian hospital and the experience statements developed for each of the SRR classes as shown in Table 6 in the associated paper.

Table A provides a view of the competency of a facility’s information infrastructure to support the technology requirements of a given set of SRRs. These SRR competency scores are then weighted by the researchers in respect to their relevancy to each of the SOC domains (Manageability, Comprehensibility and Meaningfulness), as defined by their experience statements, across each of the operational environmental domains of People, Place and Process to generate a final SOC Domain Weighted Experience Capability as shown in Table B.

A characterization of the organisational impact of these scores is detailed in Table C.

### Worked Example

#### Information capability assessment

- - A set of information capabilities were established for each of the environments of Place, People and Process. Information capabilities are characteristics that information systems require for data action in end-user services. Information capabilities come together to support and create the processes across the three operational environments:
    - People (or resources) utilizing the infrastructure (admin, patients, staff, equipment, etc.).
    - Places where the information systems are utilized (whole of hospital, specific hospital units, externally dependent campuses, car parking, etc.).
    - Processes that are dependent on the information systems (nurse call, bed management, task management, etc.).
  - The technology infrastructure to deliver information capabilities was derived from the IMA framework by taking the technology services defined within the IMA and associating them with each of the information capabilities defined within the experience framework. In doing this, a set of technology services were established for each information capability. It is important to note that a technology service could be associated with number of different information capabilities.
  - Through the IMA Framework assessment process, it has been possible to quantify the maturity of each of the technology services and these were then assigned to a level on the 4-step scale (0 to 3) of the information capability framework (see Table 4 in the main paper). The information capability framework scores from all the technology services for a specific information capability were averaged to give a score out of 3 (See Table A). This column in Table A represents the technology services IMA score translated from the IMA 8-level scale to the. information capability maturity 4-level scale. The data has come from previous IMA studies.
  - To simplify the assessment process for each information capability, the technology services were divided into two groups
    - **Common technology services:** Technology services that contribute to all information capabilities within an operational environment (people, place, and process) in the same way. A single capability assessment could be used for these services as shown in second column of Table A.
    - **Specific technology services:** Technology services whose contribution varies between the information capabilities within an operational environment (people, place, and process). The contribution of each of these technology services needed to be separately weighted for each technology service within each information capability, as shown in the third column of Table A.
    - **Total Information Capability Score:** A single information capability measure was created by averaging the common and specific information capability measures as shown in the fourth column of Table A.

#### Contribution of information capabilities to SRRs

- The next step in the framework is the critical point at which the clinical and operational teams within the healthcare facility look at the experience value statements they have created for their organisation at the SRR level, and identify the processes they have, or aspire to have, in place to achieve those experience statements. Understanding these critical processes allow the teams to weight the relevancy of each of the information capabilities and so understand their ability to support these key processes.
- As an example, the outcome of this process for a typical highly functional digital hospital is shown in columns 5 to 16 of Table A. This process yields an SRR Class Maturity score for each SRR class across each of the operational environments of People, Place and Process (note that the example in Table A is for the Process operational environment)

Table A: Output of worked example of establishing the Information Process Capabilities (how competent is the facility’s infrastructure) for each of the Specific Resistance Resource (SRR) Groups. This is done for each of the operational environments of People, Place and Process. This example is for the Process operational environment.

| **Information Capability Level** | | | | **Specific Resistance Resource Class** | | | | | | | | | | | |
| --- | --- | --- | --- | --- | --- | --- | --- | --- | --- | --- | --- | --- | --- | --- | --- |
| Information Capability | Common Technology Services contribution | Specific Technology Services contribution | Information Capability Score | Teaming and Sharing | | | Scheduling and Coordinating | | | Educating and Training | | | Monitoring and Reporting | | |
|  |  |  |  | Relevancy Weight | Weighted Capability | Weighted Contribution | Relevancy Weight | Weighted Capability | Weighted Contribution | Relevancy Weight | Weighted Capability | Weighted Contribution | Relevancy Weight | Weighted Capability | Weighted Contribution |
| Interoperating | 1.64 | 0.78 | 1.21 | 5 | 6.03 | 20.09% | 5 | 6.03 | 17.73% | 4 | 4.82 | 15.07% | 4 | 4.82 | 16.08% |
|  |  |  |  |  |  |  |  |  |  |  |  |  |  |  |  |
| Contextualizing | 1.64 | 1.13 | 1.38 | 4 | 5.52 | 18.41% | 4 | 5.52 | 16.24% | 4 | 5.52 | 17.26% | 3 | 4.14 | 13.81% |
|  |  |  |  |  |  |  |  |  |  |  |  |  |  |  |  |
| Orchestrating | 1.64 | 1.03 | 1.33 | 4 | 5.33 | 17.76% | 4 | 5.33 | 15.67% | 3 | 4.00 | 12.49% | 4 | 5.33 | 17.76% |
|  |  |  |  |  |  |  |  |  |  |  |  |  |  |  |  |
| Scheduling | 1.64 | 0.77 | 1.20 | 2 | 2.41 | 8.02% | 5 | 6.01 | 17.69% | 4 | 4.81 | 15.03% | 3 | 3.61 | 12.03% |
|  |  |  |  |  |  |  |  |  |  |  |  |  |  |  |  |
| Simplifying | 1.64 | 1.11 | 1.38 | 3 | 4.13 | 13.75% | 4 | 5.50 | 16.18% | 5 | 6.88 | 21.49% | 3 | 4.13 | 13.75% |
|  |  |  |  |  |  |  |  |  |  |  |  |  |  |  |  |
| Informing | 1.64 | 0.98 | 1.31 | 4 | 5.24 | 17.47% | 3 | 3.93 | 11.56% | 5 | 6.55 | 20.47% | 5 | 6.55 | 21.84% |
|  |  |  |  |  |  |  |  |  |  |  |  |  |  |  |  |
| Tasking | 1.64 | 0.75 | 1.19 | 3 | 3.58 | 11.93% | 5 | 5.97 | 17.55% | 3 | 3.58 | 11.19% | 3 | 3.58 | 11.93% |
|  |  |  |  |  |  |  |  |  |  |  |  |  |  |  |  |
| Trusting | 1.64 | 1.05 | 1.34 | 5 | 6.72 | 22.42% | 4 | 5.38 | 15.82% | 4 | 5.38 | 16.81% | 5 | 6.72 | 22.42% |
|  |  |  |  |  |  |  |  |  |  |  |  |  |  |  |  |
|  |  | **Information Capability Weighted SRR Maturity** | |  | **1.30** |  |  | **1.28** |  |  | **1.30** |  |  | **1.30** |  |
|  |  |  | |  |  |  |  |  |  |  |  |  |  |  |  |

With an understanding of technological strength of an organisation’s SRRs, the final issue is understanding the relevancy of the SRRs to achieving the experiences that the organisation aspires to deliver. This provides the link between technology and experience. It is achieved through taking the experience statements that have been derived through discussion and analysis with the organisation’s clinical staff, operational staff and patients, and ranking the relevancy of the SRR groups. In this case there are four categories of SRRs: Teaming and Sharing, Scheduling and Coordination, Educating and Training, and Monitoring and Reporting. The relevancy for each of the SRRs to support the desired experience statements within the SOC domains of Manageability, Comprehensibility and Meaningfulness, is estimated using the relevancy ranking in Table 8 in the associated paper and an extract of the calculation process, in this case for the SOC of Manageability and the SRR of Teaming and Sharing can be found in Table B.

Table B Example of the weighting of information maturity to relevancy in delivering an experience using Manageability.

|  | | | **Teaming and Sharing Information Contribution**. | | |
| --- | --- | --- | --- | --- | --- |
| **SOC Domain Experience Statements** | | | **Information Capability Weighted Score Average** | **SOC Domain Experience Weighting** | **Component Weighting** |
| **Manageability** The experience of managing day-to-day physical realities; staying warm, dry, clean, rested, and nourished | **Place** | * I can influence or control the environment * I have sufficient information about the healthcare environment to form reasonable expectations * The environment provides sufficient amenities and facilities to reduce stress and enhance wellbeing. I feel more able to be in a positive mood because of an environment tailored to my personal preferences * The environment is designed in a way that builds reliability. My environmental needs will be taken seriously. The environment is uniform and consistent with my specified requirements. It is responsive to my needs. | 1.33 | 4 | 5.34 |
|  | **People** | * The people I engage with respect my perspective, culture and values in the interactions we have  * There are key people who I know to go to during my care, and they are responsive to my needs * I am confident that I can engage with my team to revise, change or strengthen my care goals whenever I feel appropriate. * The people I meet during my care are reliable, competent and trustworthy. | 1.37 | 5 | 6.84 |
|  | **Process** | * I can tailor aspects of my care within the larger process of a health organisation  * The demands of the process are reasonable and allow for choices and the needs of my life outside the health organisation. * Tension is reduced because the process is efficient and effective and conforms to my evolving needs * The processes are knowable, reliable, and effective, and I have developed confidence in them. | 1.30 | 3 | 3.90 |
| **SOC Domain Weighted Experience Capability** | | | | | **1.34** |

The SOC domain weighted experience capabilities from each of the SRRs can be aggregated across the four SRRs to generate a single score out of 3.00 that represents the capability of the information infrastructure to support the desired experiences. This single number for each of the SOC domains (Manageability, Comprehensibility and Meaningfulness) has been categorized into four levels which are described in information infrastructure terms in Table C. The technology score reflects the competency of the information infrastructure to support a given level of experience within each of the SOC domains of Manageability, Comprehensibility and Meaningfulness.

Table C. Information Infrastructure Experience Capabilities.

| Technology Score | Experience Definition | Description |
| --- | --- | --- |
| 0.00-0.75 | **Unsatisfactory** Information technology does not play a significant role in creating experiences within the organisation | The information infrastructure at the unsatisfactory level is insufficient to reliably support the systemic reduction of environmental stressors for either patients or staff. The information infrastructure will largely lack the capabilities to deliver a direct and sustainable high-quality patient experience. These shortfalls reflect an environment that does not consistently support an individual’s Sense of Coherence. The environment has a low, or imbalanced, set of capabilities, which do not support a patient’s sense of manageability, comprehensibility, and meaningfulness. These outcomes are impacted by an information infrastructure that is deficient in many technical capabilities forcing both the clinician and patient to depend on extensive ad hoc interventions to reduce the environmental factors contributing to an individual’s stress. For example, a patient may feel that they are not fully understood, lack transparency, and have little control over their situation. There is reduced trust in the decision-making process and the information provided. |
| 0.76-1.50 | **Engaged**  This is a task-oriented level where the information-driven experiences are centred around the availability, reliability, and relevancy of the information   (The experiences are based on the amalgamation of the Information Capability Level 1 ‘Information’ experiences across the IPCs for Place, People and Process) | This level is characterized by the potential variability in information technology performance. This variability may not support the three Sense of Coherence Domains of Measurability, Comprehensibility and Meaningfulness. The potential uneven contribution to the domains needs to be accounted for when interpreting the results. In a well-balanced information infrastructure with matching contributions of each of the SOC domains, this level has improved capability to define an individual’s environment and the characteristics of people and places they need to interact.  Examples of relevant information include individual’s diagnostic information, staff and patient location, optimum staff and patient contact processes, equipment availability, skills and skillset availability, process status information (e.g., test and support services availability).  This type of information enables patients to understand better their situation and those of others they need to work with. It provides the information foundations to understand how best to work with others and optimum methods for interaction. The information provided to patients and staff is clearly articulated and structured to be appropriate for the user’s skill levels and culture. This level provides the foundation for delivering task-level activities that require complete and accurate, and interpretable information from the environment and the individuals within that environment. |
| 1.51-2.25 | **Transformed** This is a process-oriented level that enables tasks to be assembled into processes to deliver common objectives. The information-driven experiences are centred around creating cooperation between people, systems and the interface between people and systems   (The experiences are based on the amalgamation of the Information Capability Level 2 ‘cooperation’ experiences across the IPCs for Place, People and Process) | An increased uniformity characterises this level in the information infrastructure performance to support the three Sense of Coherence Domains of Measurability, Comprehensibility and Meaningfulness. However, potential variances should be considered when interpreting these results. In a well-balanced information infrastructure with comparable contributions of each of the SOC domains, this level provides the ability to bring together individuals into groups to enable the more effective delivery of services and the support of the patient. This level provides the capability to mold the processes that individual interacts with. It provides for the rapid and convenient sharing of information in a way appropriate for the skill level and culture of the individual consuming it. It provides the patient with enhanced capabilities to influence their care process and the characteristics of their environment. The clinician feels more effective in their decision making based on optimized information at the point of care. The patient feels that they can fully engage with the clinician based on optimized information that is transparent but secure. There is moderate trust in providing privacy and safety. |
| 2.25-3.00 | **Sustained** This is a systems-oriented level where process outcomes are linked to create and manage complex systems. The information-driven experiences centre around arranging processes in both time and geography to simplify and automate processes making them robust and responsive to the needs of patients and staff, leveraging the evolving capabilities of the facilities they exist in.   (The experiences are based on the amalgamation of the Information Capability Level 3 ‘Systemization’ experiences across the IPCs for Place, People and Process). | This level is characterized by a high degree of uniformity in the information infrastructure performance to support the three Sense of Coherence Domains of Measurability, Comprehensibility and Meaningfulness. At this level, the information infrastructure enables complex processes to form into systems around both the patient and the staff to enable the delivery of clinical services that are effective, robust, and responsive to the changing needs of the individual. The patient's system interacts with has a high degree of potential automation that can be applied as appropriate. The clinical and operation systems are transparent to and influenced by the individual. Patients and staff have a clear understanding of their situation and optimise their clinical and operational engagement. They are well supported both clinically and socially and feel a low level of personal stress. They have a high level of trust in the information provided. |
